# Recent advances in predicting gene–disease associations

**DOI:** 10.12688/f1000research.10788.1

**Published:** 2017-04-26

**Authors:** Kenneth Opap, Nicola Mulder

**Affiliations:** 1 University of Cape Town, Cape Town, South Africa

**Keywords:** gene-disease association, Genome Wide Association Studies, GWAS, linkage anaylsis, computational prediction

## Abstract

Deciphering gene–disease association is a crucial step in designing therapeutic strategies against diseases. There are experimental methods for identifying gene–disease associations, such as genome-wide association studies and linkage analysis, but these can be expensive and time consuming. As a result, various *in silico *methods for predicting associations from these and other data have been developed using different approaches. In this article, we review some of the recent approaches to the computational prediction of gene–disease association. We look at recent advancements in algorithms, categorising them into those based on genome variation, networks, text mining, and crowdsourcing. We also look at some of the challenges faced in the computational prediction of gene–disease associations.

## Introduction

Aberrations in certain genes have been observed to either predispose individuals to disease or be directly responsible for the development of a disease phenotype, as in the case of Huntington’s disease ^1^ and sickle cell disease ^2^. Deciphering the link between genes and diseases is an open problem in biomedical sciences, but it presents an opportunity to better understand disease aetiology, thereby allowing for the design and development of better mitigation strategies. Note, here we are describing only the links or associations between genes and disease rather than suggesting causality, as the issue of causality is still under debate.

Experimental methods for gene–disease association, such as linkage studies ^3^, genome-wide association studies (GWAS) ^4^, and RNA interference screens ^5^, are expensive and time consuming to run. As a result, a number of computational methods ^6– 8^ have been developed to identify or predict gene–disease associations. These methods have different strengths and weakness and are suited for different classes of disease. For instance, methods that are suited for monogenic diseases, such as those that look at candidate disease gene expression patterns, may perform poorly when applied to a complex disease whose aetiology is attributed to many genes that work in concert to elicit the disease phenotype. In complex diseases, the genes that are responsible for disease phenotype when individually investigated are often found to give signals too weak to assign gene–disease association. One such example, as suggested by GeneRank ^9^, is the case where genes that are strong drivers of disease are transcription factors that may not be differentially expressed between disease and non-disease conditions but are responsible for regulating the expression of other genes that are differentially expressed.

The diversity of data that is used to derive gene–disease relationships as outlined in the review of tools in 6– 8 is a clear testament to the complexity of biological systems. Consequently, methods that incorporate diverse data sets, such as that described in 10, tend to achieve better results for the reason that when a gene–disease association is backed by many heterogeneous methods and data, it is more likely to be a true association. In deriving gene–disease associations, different tasks can be performed in parallel or as part of a sequential pipeline. Some of the activities required include combinations of the following:

- Identifying variants that are associated with the disease and identifying genes that are associated with the variants.
- Establishing gene–disease association via other methods. In some cases, gene–disease association is derived from differential expression of genes in disease and non-disease conditions. Text mining biomedical literature is also a very popular source of gene–disease association data for most computational tools owing to the fact that the data are relatively easy to access. However, the success of text mining methods is heavily dependent on the quality of the text data and the efficiency of the algorithms.
- Assigning some confidence to the established gene–disease association, e.g. assigning weights based on where the association was derived from (experimentally derived, expertly curated, or predicted from text).
- Identifying publications that support the association. Some tools use publication support as a preliminary step in retrieving candidate disease genes. Often tools that use text mining as a basis for assigning gene–disease associations retrieve co-mentions of genes and diseases from biomedical literature when drawing a pool of candidate genes, which are further examined for association with a given disease. In other cases, the number of publications that support a particular gene–disease association is used as a basis for ranking the validity of the association.
- Presenting and distributing the results, which addresses the format in which the data are presented and distributed. Currently, data representation in scientific research is geared towards satisfying two key needs: a) that the data can be easily accessed and interpreted by non-technical users for the purposes of knowledge acquisition and b) that the data are accessible to technical users for the purposes of extending the tool, e.g. application programming interfaces (APIs), or for large-scale data analysis.

Accordingly, tools are being developed to address each of the components above. Some tools amalgamate two or more components into one contiguous process that is packaged into a single tool. In some cases, the whole gene–disease association discovery engine is infused into a single platform, such as in the case of DisGeNET ^11^.

This article seeks to review recent advances in elucidating gene–disease associations by investigating strengths of current computational methods and some of the challenges. The list of the tools that we review is by no means exhaustive, but we focus on some tools that have used innovative ways to advance gene–disease association algorithms. We have categorised the tools based on the approach used—1) genome variation, 2) text mining, 3) crowdsourcing, and 4) networks—and provide some examples of each. Summary information for the examples is provided in Table 1.

**Table 1.** A brief summary of some of the tools that have been reviewed in this article. Each tool is classified according to the categories that are described in the introduction section, the algorithm used, the technology used in implementation, the data sources used, and how the tool can be accessed.

Tool	Algorithm	Technology	Data Sources	Accessibility
**Variation** DisGeNET	GWAS	Python, R, Bash, SPARQL	CTD Uniprot ClinVar OrphaNet GWAS catalogue RGD MGD GAD BeFree	Cytoscape app RDF SPARQL endpoint Scripts (Python, R, Perl, Bash) R Package Linked open Data cloud
**Text Mining** MOPED-Digger Inductive Matrix Completion Implicitome	NLP (co-occurrence of gene–disease in abstracts) Matrix completion NLP (Peregrine)	Java, Apache Lucerne C/C++, Python, MATLAB Java	PubMed OMIM UMLS Entrez Gene OMIM Uniprot HGNC JoChem	Desktop application Desktop application Desktop application
Reference Variant Store (RVS)	Variant annotation, data integration	Apache Hadoop Python, Java, JavaScript, Scala, MySQL	1000 Genomes EXAC Scripps Wellderly	RESTful APIs Web
**Crowdsourcing** Dizeez	Text mining tools, the crowd (MTurkers)	Java, Perl, C++, web technologies	OMIM PubMed PubChem	Web
**Networks** HeteSim Multipath (HSMP)	Support vector machine, multipath analysis	MATLAB	OMIM, HumanNet, HPRD	Desktop application

API, application programming interface; CTD, Comparative Toxicogenomics Database; EXAC, Exome Aggregation Consortium; GAD, Genetic Association Database; GWAS, genome-wide association studies; HGNC, Human Genome Organisation (HUGO) Gene Nomenclature Committee; HPRD, Human Protein Reference Database; MGD, Mouse Genome Database; NLP, natural language processing; OMIM, Online Mendelian Inheritance in Man; RDF, resource description framework; RGD, Rat Genome Database; SPARQL, SPARQL protocol and resource description framework query language; UMLS, unified medical language system.

## Genome variation

GWAS and genetic linkage studies ^3^ are the main methods used for identifying variations across the genomes of individuals and associating these with diseases or phenotypes. The idea behind GWAS is to establish whether there is a significant genetic variation between case and control populations for a given phenotype under investigation.

The most common type of variation studied for diseases is the variation at a single nucleotide position, otherwise known as the single nucleotide polymorphism (SNP), although other types of variation such as copy number or chromosomal rearrangements have also been linked to many diseases. GWAS identify marker SNPs that are associated with the phenotype/trait under investigation. Once the marker SNPs have been identified, the next challenge is to determine how the variants are responsible for the phenotypes. This entails finding the location of the SNPs in relation to genes and, if associated with a gene, then identifying the pathways the gene is involved in. Genetic linkage studies, on the other hand, identify linked regions on the genomes of related individuals by observing the transmission of the loci from parents to offspring that is expected by independent inheritance. Genetic linkage is used to find regions in the genome that predispose an individual to a particular phenotype.

For *in silico* studies, the association data are usually obtained from some of the many databases that maintain genotype–phenotype information. The review of Brookes and Robinson ^12^ lists some of the databases that contain genotype–phenotype data in relation to human health. The databases contain more or less similar genome variation data; however, they differ in aspects such as the data access policies, the standards that they employ when curating the data, and the expertise of the database curators. Some databases such as Orphanet ( www.orpha.net) ^13^ and OMIM ( www.omim.org) ^14^ cater for domain-specific phenotypes, i.e. rare and Mendelian diseases, respectively, which encourages use by domain experts. However, the preference of one particular database over another largely depends on the individual requirements of the user, although some databases, such as the GWAS catalogue ( www.ebi.ac.uk/gwas/) ^15^, are widely used owing to their comprehensive coverage of variation data and ease of access. The GWAS catalogue presents the variation data in an interactive karyogram that can be easily queried by different parameters in addition to offering programmatic access to the data. These facilities encourage adoption of the resource. While dbSNP ( https://www.ncbi.nlm.nih.gov/projects/SNP/) is a commonly used source of variants, it does not attempt to cover variant-disease associations. ClinVar ( https://www.ncbi.nlm.nih.gov/clinvar/), on the other hand, provides a clinical or phenotypic association for variants, with supporting evidence from multiple sources. The Reference Variant Store (RVS) ( http://rvs.u.hpc.mssm.edu/) ^16^ is perhaps the single most comprehensive repository for genome variation data both in size (over 400 million variants and 80,000 samples) and in the variety of annotation data that are stored. The RVS also has, as one of its main features, a RESTful API for the flexible retrieval of data by different features such as frequency, prediction method, disease, and literature.

There are a number of tools that use a combination of outputs from GWAS or linkage studies, next-generation sequencing (NGS), and data from the abovementioned resources to prioritise gene–disease association. One example is Exomiser ^17^, which incorporates variant annotation, protein interaction networks, and phenotype, clinical, and other information for disease gene identification for Mendelian diseases from a variant call format (VCF) file. Algorithms have been developed to predict the effects of changes in the DNA or protein sequence based on certain properties of sequences. SIFT ( http://sift.jcvi.org/) ^18^, PolyPhen-2 ( http://genetics.bwh.harvard.edu/pph2/) ^19^, and PROVEAN ( http://provean.jcvi.org/) ^20^ are some of the tools that are used in predicting the phenotypic effects of genome variation. CADD ( http://cadd.gs.washington.edu/) ^21^ is also used in many cases for gene–disease association studies to prioritise functional, deleterious, and pathogenic variants. It works by integrating diverse annotation sources into a single *C score.*

## Text mining

The bulk of scientific knowledge is still kept in textual format, although the availability of these data in scientific databases is also growing exponentially. For instance, Burger *et al*. ^22^ estimate that articles about gene–disease associations that are deposited in public databases grow at the rate of about 10,000 papers per year (approximately one paper every hour of every day). As a result, there is an increasing need to find better and faster ways of retrieving and processing knowledge from scientific databases. Databases that are manually curated by experts provide high-quality data, albeit at a very slow pace, so text mining algorithms are now being used to automate some manual processes.

Gene–disease association may be derived from direct association of a gene with a disease in biomedical text ^23– 25^. In some cases, implicit association between genes and diseases is used, as demonstrated in 26, wherein a gene X is implicitly associated with a disease Z if it is directly associated with a biological concept (gene, drug, phenotype, or biological process) Y, which is also directly associated with the disease Z.

The National Centre for Biotechnology Information (NCBI) maintains a set of high-quality text mining software in its tool set. Some examples of tools that are relevant for processing genome variation information include tmVar ^27^, for extracting sequence variants at the levels of both genes and proteins from biomedical literature; DNorm ^28^, which is a resource that is used to automatically identify disease names in biomedical text; and GNormPlus ^24^, which identifies gene mentions and normalization in biological text. Gene normalization, as described in 29, is the process of identifying and assigning biomedical database identifiers to genes retrieved from biomedical text. In order to improve efficiency, GNormPlus integrates other resources such as SimConcept ^30^ for identifying and simplifying composite names and SR4GN ^31^ for species named entity identification in biomedical text. PubTator ^28^ is another resource for biocuration that incorporates biomedical text search. A user may search for PubMed articles by the following terms: gene, disease, PubMed, or chemical. PubTator incorporates precomputed searches from tools such as GNorm, DNorm, and SR4GN.

From the tools discussed above, a simple text mining-based gene–disease association can be implemented by performing a PubMed-like keyword search using PubTator, using normalisation and annotation tools to retrieve relationships between concepts (tmVar for mutation, GNormPlus for genes, and DNorm for diseases), and then presenting the results for visual inspection or integration into other analysis pipelines.

## Crowdsourcing

Crowdsourcing refers to the act of delegating a job traditionally assigned to a dedicated agent (usually an employee) to a large group of people in the form of an open call ^32^. The immense quantity of data that biomedical scientists need to deal with today has prompted the search for innovative ways of solving scientific problems. The following qualities identify suitable candidates for crowdsourcing solutions:

1) Few individuals with rare abilities could solve the problem. It is sometimes difficult to harness all the necessary skills for a particular task in one organization or through traditional ways of collaboration.
2) The problems are simple tasks that require human intelligence, e.g. annotating images.
3) The problems can be broken into tasks with definite endpoints. The possibility of breaking jobs into smaller tasks translates to the possibility of sharing the incentives with a larger group of people and, in essence, simplifying the problem.

Many problems in bioinformatics possess the qualities listed above, and some scientists have explored the use of crowdsourcing methods to solve these problems ^33^. Researchers design tasks for which they wish to recruit a crowd and then invite workers to participate in the tasks by using crowdsourcing platforms such as Crowdflower ( http://www.crowdflower.com), Amazon Mechanical Turk (AMT) service ( https://www.mturk.com), and Kaggle ( www.kaggle.com).

Several crowdsourcing approaches have been used to identify gene–disease associations. Dizeez ^34^ works as a multiple quiz game in which a player is presented with a disease drawn from the Human Disease Ontology ^35^ as the “clue” and a list of five genes. Only one of the five genes has been linked to the clue disease before. The player is challenged to accumulate points by guessing the correct gene–disease links. All guesses are taken as “assertions” and examining the frequencies of the “assertions” for unknown links identifies new gene–disease associations. Running simulations in which a player randomly assigned gene–disease associations validated the results of Dizeez by showing that there was a significant difference with the real results from playing the game. In another approach, Burger *et al*. ^22^ adopted a hybrid method in which they used gene and mutation tagging tools GenNorm ^29^ and Extraction of Mutation (EMU) ^36^, respectively, to extract gene-mutation pairs from PubMed abstracts. Each gene-mutation pair is then presented to the recruited workers in the AMT service as a human intelligence task (HIT) *.* Basically, a HIT according to 22 is a minimal task that cannot be automated. The quality of the crowdsourced service is evaluated by redundancy and aggregation in such a way that the same task is presented to five different workers and the congruency of their results is evaluated, the idea being that a result that is supported by many workers is most likely to be correct. Like in Burger *et al*. ^22^, Li *et al*. ^37^ also incorporated text mining tools tmChem ^38^ and DNorm ^28^ in addition to the wisdom of the crowd to identify associations between chemical substances and diseases from text.

The review articles 26 and 32 together with 39 provide more information on crowdsourcing in biomedicine, particularly touching on how to choose the right crowdsourcing platform for a particular task and some of the challenges that one may face when using crowdsourcing to solve problems in bioinformatics.

## Networks and semantic similarity-based algorithms

Network algorithms rely on the premise that phenotypically similar diseases are caused by genes that are functionally related ^40^. The idea is to find a set of genes that are already linked to the disease or phenotype in question and then find genes that are functionally related to that set. Many examples of network-based methods have been reviewed in Piro & Cunto ^6^ and two are mentioned below. HeteSim ^41^ integrates heterogeneous networks of protein–protein interaction (PPI), gene–phenotype association, and phenotype–phenotype similarity to prioritise novel gene–phenotype associations. Natarajan & Dhillon ^42^ formulate the gene–disease association problem in a similar way to a recommendation problem in which the players are genes as the “recommenders”, ** and diseases are the “items” ** that they recommend or “prefer”. The goal is to identify which diseases a given set of genes would prefer given a set of observed preferences provided as biological entities.

## Discussion

Gene–disease association is a crucial step in understanding disease aetiology. The process has been directed by manually curated biomedical databases owing to the faith that is placed on expert knowledge and individual attention. The exponential rate at which biomedical databases grow is quickly rendering manual curation of biomedical databases unattainable. The big challenge now is that of obtaining gene–disease associations on a large scale while at the same time not compromising on the quality of the associations. Scientists have developed innovative solutions in trying to solve this problem, ranging from adapting popular algorithms from other fields, like in the case of GeneRank adapting Google’s PageRank ^9^, to using crowdsourcing platforms ^22, 34^.

From the tools discussed above, a common trend is that most gene–disease association tools are built in a modular manner such that different standalone components are aggregated together to form the complete tool. For example, a tool that identifies mutations in biological text like EMU ^36^ can be combined with a tool that performs gene normalisation like GenNorm ^29^ to build a mutation-finding tool like that of Burger *et al*. ^22^. One of the challenges is standardisation of the data across the tools while still maintaining quality, especially when the different data sources are constantly updated. One would need to determine whether the different components are using the same database version. A solution would be to use third-party data providers such as CellBase ^43^, which provides web services for retrieving biological information from heterogeneous sources to handle data harmonisation across different tools.

Unconventional approaches such as crowdsourcing gene–disease association have also helped to partially deal with the inherent problem of volume and quality control of data that are saved into the databases. Redundancy and aggregation is one of the chief quality control methods that is employed by many crowdsourcing projects in bioinformatics ^33^ owing to the availability of a large pool of experts willing to work for relatively affordable compensation, even for free in some cases.

Another observation about the methods described is that although the algorithms are hardly altered—for example, network algorithms still look for functional links among genes and text mining algorithms still parse biological text in order to unearth relationships between genes and diseases—the innovation is in the implementation of the algorithms and in handling some of the inherent weaknesses of the algorithms such as limited data. As an illustration, the crowdsourcing algorithm in Burger *et al*. ^22^ substitutes human labour for tasks that would otherwise be performed by software. Another example is the transferring of annotation between different but related biological components to complement limited data, like in the case of a literature-wide association study (LWAS) that is applied in Implicitome ^26^. In Implicitome, a connection between a gene and a disease is obtained by independently mining literature for a connection between a gene and a biological component, which, in turn, has literature that links it to a disease.

Another recurrent theme in this review is the integration of different modules and data sources, whether as a distinct part of an algorithm or integration of similar data to ensure comprehensive coverage. This requires the addressing of the issues of compatibility and standardisation so that different components can link harmoniously. Many tools make use of ontologies such as the disease ^35, 44^ and phenotype ontologies ^45^ for data standardisation.

## Challenges

The two biggest challenges in gene–disease associations are how to store and display the relevant data for retrieving gene–disease associations in a readily accessible manner for researchers with varying levels of technical expertise and scalability of algorithms. As mentioned previously, standardisation of data across different platforms is important, but so are considerations of how to deal with controlled access. The development of software that scales with the rate of increase in data size and complexity is also a major challenge. How do you build efficient software that will incorporate the changes in knowledge both in a timely manner and on a large scale? A third challenge is the integrity of the resulting associations and attributing evidence to assertions made by algorithms. While gene–disease associations can improve our knowledge on disease aetiology, it is still an area of active research and these associations should not be used in a clinical setting without further validation. Environment and context can have an important effect on the impact and relevance of a gene– (or variant)–disease association, so the data cannot be used in isolation.

There are many groups working globally on gene–disease associations in terms of method development, data consolidation, or experimental versification, and only a few are mentioned in this review. The Global Alliance for Genomics and Health ( http://genomicsandhealth.org/), for example, has genotype to phenotype and variant interpretation projects, and many of the cancer initiatives focus on the clinical interpretation of variants. Here we have focussed only on some of the recent methods for predicting gene–disease associations to provide a taste of the different approaches.

## Data sources

Listed below are some of the data sets that are used by tools that we reviewed.

**OMIM** ( www.omim.org): Online Mendelian Inheritance in Man ^46^

**CTD** ( http://ctdbase.org/): The Comparative Toxicogenomics Database—provides data about interactions between chemicals and gene products and how the interactions are related to diseases ^47^

**ClinVar** ( https://www.ncbi.nlm.nih.gov/clinvar/): an archive for interpretations of the clinical significance of genetic variants ^48^

**OrphaNet** ( www.orpha.net): an online rare disease and orphan drug database ^13^

**The GWAS Catalog** ( www.ebi.ac.uk/gwas/): manually curated, quality-controlled, literature-derived database of GWAS ^15^

**MGD** ( http://www.informatics.jax.org/): the Mouse Genome Database ^49^

**RGD** ( http://rgd.mcw.edu/): the Rat Genome Database ^50^

**LHGDN** ( http://www.dbs.ifi.lmu.de/~bundschu/LHGDN.html): the literature-derived human gene-disease network—text mining-derived database for classifying gene–disease associations

**BeFree** ( http://ibi.imim.es/befree/): gene–disease associations extracted from MEDLINE abstracts using BeFree system ^51^ for text mining

**GAD** ( https://geneticassociationdb.nih.gov/): the Genetic Association Database, which is an archive of complex diseases in humans ^52^

**ExAC** ( http://exac.broadinstitute.org/): the Exome Aggregation Consortium, which collects and harmonises exome sequencing data from large exome sequencing projects ^53^

**HGNC** ( http://www.genenames.org/): the HUGO Gene Nomenclature Committee, which is a database for human gene names and symbols ^54^

**JoChem** ( http://biosemantics.org/index.php/resources/jochem): a dictionary to identify small molecules and drugs in text ^55^

## Abbreviations

AMT, Amazon Mechanical Turk; API, application programming interface; EMU, Extraction of Mutation; GWAS, genome-wide association studies; HIT, human intelligence task; RVS, Reference Variant Store; SNP, single nucleotide polymorphism.
